# Tracheostomal Myiasis: A Case Report and Review of the Literature

**DOI:** 10.1155/2011/303510

**Published:** 2011-12-20

**Authors:** S. Prasanna Kumar, A. Ravikumar, L. Somu, P. Vijaya Prabhu, Rajavel Mundakannan Subbaiya Periyasamy Subbaraj

**Affiliations:** Department of ENT, Head and Neck Surgery, Sri Ramachandra Medical College and Reseach Institute, Porur, Chennai 600116, India

## Abstract

“Myiasis” is considered in Hindu mythology as “God's punishment for sinners.” It is known to infest live human or animal tissue. Literature abounds with reports of myiasis affecting the nasal cavity, ear, nonhealing ulcers, exophytic malignant growth, and cutaneous tissue. But report of myiasis of the tracheal stoma is rare. Only a few cases of tracheal myiasis have been reported in literature. We report a case of tracheostomal myiasis in an elderly male. The species which had infested the stoma was identified as *Chrysomya bezziana*, an obligate parasite. This is to our knowledge the first case report of an obligate parasite (*Chrysomya bezziana*) infestation of the tracheostoma from India.

## 1. Introduction

Hindu mythology describes myiasis as “God's punishment for sinners” [1, 2]. One of the earliest reports was from Soares “d” Souza (1587) who reported a case of cutaneous myiasis [2]. In 1840, Rev F. W. Hope coined the term “myiasis” (earlier known as scholechiasis). Castellani and Chalmer's (1919) described nasal myiasis caused by chrysomya [2] known as Peenash in India. Myiasis infesting various parts of the body abounds in the literature; however, we could find only four cases of myiasis of the tracheal stoma that have been reported. We report a rare case of an obligate parasite which had infested the tracheal stoma and discuss the successful management along with review of the literature.

## 2. Case Report

**Figure 1:**
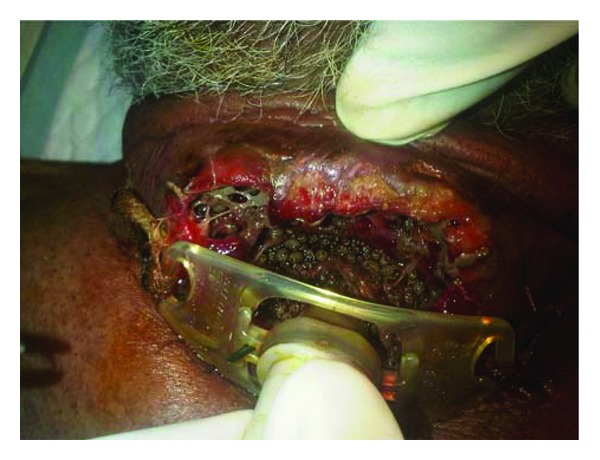
Maggots seen around the tracheostoma.

**Figure 2:**
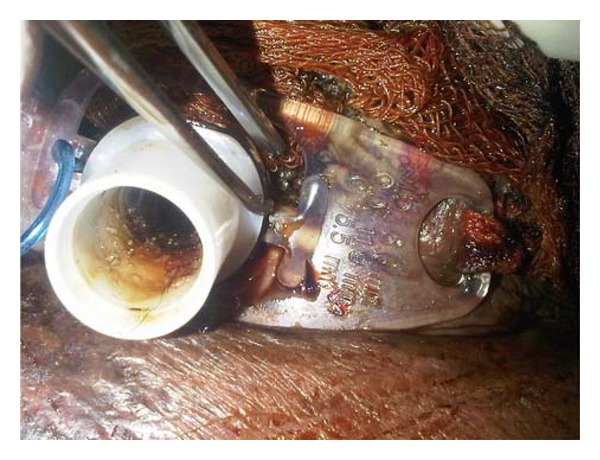
Maggots being removed from around the stoma.

**Figure 3:**
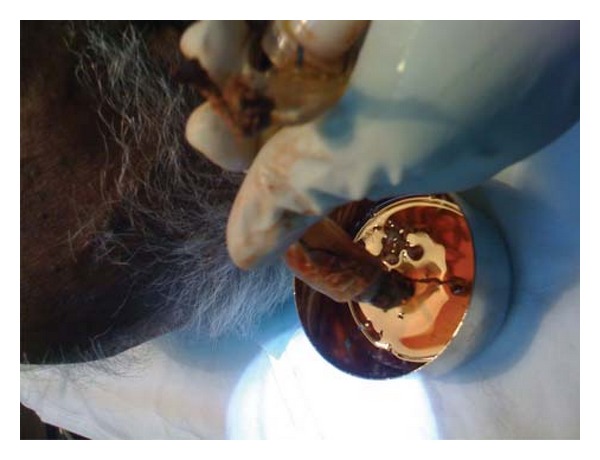
The blocked tracheostomy tube at presentation.

**Figure 4:**
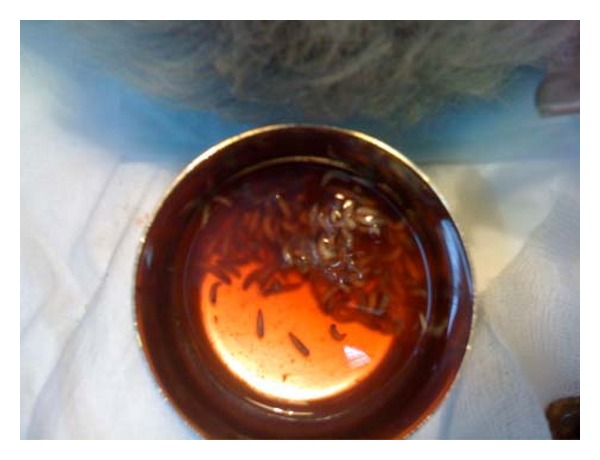
Maggots removed on day 1.

A 78-year-old, tracheostomised patient came to our emergency department with respiratory distress. He had noisy laboured breathing and had foul smelling blood-stained discharge from around the tracheostomal site. Examination showed maggots creeping all around the tracheostomy tube. Portex-cuffed tracheostomy tube (size 8.0) was almost fully blocked with secretions, crusts, and maggots (Figures 1, 2, and 3). Adequate precautions were taken, and tracheostomy tube was changed. About 100 live maggots were removed carefully from around the tracheostoma (Figure 4). The patient was stabilised and admitted in isolation ward. Multiple wound debridements with removal of maggots using turpentine soaked gauze were done, taking adequate precautions so that turpentine was not aspirated. The patient was administered intravenous antibiotics, and tracheostomy tube care protocol was followed. In total, 230 maggots were removed over the span of 72 hours. Wound swab culture from the wound grew *E. coli, Serratia marcescens,* and *Enterococcus Faecalis*. After 72 hours, there were no more maggots, and the tracheostomal wound started healing well. By day six of admission, the wound healed well. Patient was discharged in a stable condition with Shiley tracheostomy tube (size 6).

This patient had earlier undergone tracheostomy 2 years back, for head injury sustained after a road traffic accident. Decannulation had been attempted twice in the past elsewhere but had failed.

The maggots were white in colour and 10 to 12 mm in length. These maggots were sent for entomology review and were identified as *Chrysomya bezziana* [3].

Patient was followed up after 2 weeks. Shiley's tracheostomy tube was in situ. Tracheostomy wound was found to be healthy. He was advised regular followup. His care takers were educated about tracheostomy tube home care.

## 3. Discussion

Zumpt (1965) defined “myiasis” as “the infestation of live human and vertebrae animals with dipterous larvae, which at least for certain period feeds on the host's dead or living tissue liquid body substances, or ingested food.”

 Myiasis causing larvae is either an obligate parasite or a facultative parasites [1–4]. Dipterous larvae in the obligate group develop in living tissue of the host, and this is a necessary part of their life cycle. In contrast, facultative group consists of species that are free living, feeding on decaying material, that is, animal carcasses [4].

*Chrysomya bezziana*, also known as “old world screwworm,” is an obligate parasite and belongs to the order Diptera, family Calliphoridae, and suborder Cyclorrhapha. The adult fly of *Chrysomya bezziana* is a green or blue-green fly that is widely distributed in tropical and subtropical countries of Africa and Asia, including Southeast Asia, India, Saudi Arabia, Indonesia, the Philippines, Papua, New Guinea, and Persian Gulf [5, 6]. The adult fly feeds on decomposing corpses, decaying matter, excreta, and flowers. Adult female fly lays eggs only on live mammalian tissue, depositing about 200 eggs at sites of wound or in body orifices such as ear and nose. The development of *Chrysomya bezziana* from egg to adult fly can be completed in 18 days under optimal conditions. The eggs hatch after 12–18 hours, and the first-stage larvae, white in colour and 1.5 mm in length, will emerge from the eggs and then burrow gregariously, head downward into wound or wet tissues in characteristic screw worm pattern. The larvae are unable to develop in carrion. They feed on the living tissues, and the wounds increase in sizes as they feed. In about four days, the larvae moult into the second and third stages, 4–18 mm in length. After 5–7 days, the third-stage larvae would leave the wound and fall to the ground to pupate, transformed into adult fly around seven days later.

Vegetative state of the patient, psychiatric illness, immunocompromised individuals, exposed wound with foul smelling discharge, infective dermatitis, Hansen's disease, low socioeconomic status, close proximity to domestic, and peridomestic animals such as dogs and rats [7] are few of the predisposing factors for myiasis.

We could find only a handful of cases reported in literature. In one case, the myiasis was secondary to an aspirated foreign body via the tracheostomy tube lodged in the intrathoracic trachea [8]. Two cases have been reported in patients who had a tracheostomy for thyroid malignancy [7, 9]. Others have reported myiasis around the tracheostoma in a patient who was in persistent vegetative state [1, 10].

The main stay of treatment is removal of maggots with thorough wound debridement along with management of systemic and comorbid illness.

 Larvae can also be killed by applying proper insecticides to the infected areas and making sure the wounds are properly dressed. Organophosphorus insecticides like coumaphos, dichlofenthion, and fenchlorphos can be applied to wounds with fly larvae [11]. Ether, chloroform, and turpentine oil can be used to suffocate the larvae. These cause the larvae to leave the wound and fall to the ground, and the larvae will die without a host to feed on. Another method that has been tried is the use of single dose of subcutaneous ivermectin (200 microgram/kg) or doramectin (200 microgram/kg), which prevents strike and restrike of treated wounds [12–14]. Risks of use of organophosphorous compounds for tracheostomy wound myiasis is high and hence not used.

We have been using turpentine oil for all cases of myiasis in the ENT region with success, but a word of caution is that while using it around the tracheostoma, if adequate precaution is not taken, there is a high risk of chemical pneumonitis.

We need to be aware of a few problems that can occur with maggots around the tracheostomy tube. Aspiration of the maggots themselves is a major concern causing airway obstruction and aspiration pneumonitis. Aggressive nonjudicious removal of maggots in the neck can also damage the major blood vessels of the neck (carotid artery and jugular vein) causing torrential bleeding. Embolisation of maggots via the blood vessels is an expected complication too, causing thrombosis of the adjoining vessel.

## 4. Conclusion

Myiasis of the tracheostomy wound is extremely rare, with few cases reported, this is the first reported case of obligate parasite *Chrysomya bezziana* infestation of the tracheostomy wound.

Management of myiasis of the tracheostomy wound requires utmost precautions to prevent aspiration of maggots and the chemicals used to remove maggot.

Though infestation of the tracheostoma with maggots is rare, such a possibility exits. Otorhinolaryngologists need to be aware of this condition. We emphasize the importance of health education in home tracheostomy tube care to the patient and his family which will go a long way in preventing such an adverse event from occurring.
